# Effectiveness and safety of tourniquet utilization for civilian vascular extremity trauma in the pre-hospital settings: a systematic review and meta-analysis

**DOI:** 10.1186/s13017-024-00536-9

**Published:** 2024-03-19

**Authors:** Ying-Chih Ko, Tou-Yuan Tsai, Chien-Kai Wu, Kai-Wei Lin, Ming-Ju Hsieh, Tzu-Pin Lu, Tasuku Matsuyama, Wen-Chu Chiang, Matthew Huei-Ming Ma

**Affiliations:** 1 Institute of Epidemiology and Preventive Medicine, College of Public Health, National Taiwan University, Taipei, Taiwan; 2 Section of Emergency Medicine, Department of Medicine, National Taiwan University Cancer Center, Taipei, Taiwan; 3 Department of Emergency Medicine, National Taiwan University Hospital, Taipei, Taiwan; 4 Emergency Department, Dalin Tzu Chi Hospital, Buddhist Tzu Chi Medical Foundation, Chiayi, Taiwan; 5 School of Medicine, Tzu Chi University, Hualien, Taiwan; 6 Institute of Health Data Analytics and Statistics, College of Public Health, National Taiwan University, Taipei, Taiwan; 7 Department of Emergency Medicine, Kyoto Prefectural University of Medicine, Kamigyo-ku, Kyoto, Japan; 8 Department of Emergency Medicine, National Taiwan University Hospital Yun-Lin Branch, Yunlin, Taiwan

**Keywords:** Vascular extremity trauma, Tourniquet, Pre-hospital, Emergency medical service

## Abstract

**Background:**

Tourniquets (TQ) have been increasingly adopted in pre-hospital settings recently. This study examined the effectiveness and safety of applying TQ in the pre-hospital settings for civilian patients with traumatic vascular injuries to the extremities.

**Materials and methods:**

We systematically searched the Ovid Embase, PubMed, and Cochrane Central Register of Controlled Trials databases from their inception to June 2023. We compared pre-hospital TQ (PH-TQ) use to no PH-TQ, defined as a TQ applied after hospital arrival or no TQ use at all, for civilian vascular extremity trauma patients. The primary outcome was overall mortality rate, and the secondary outcomes were blood product use and hospital stay. We analyzed TQ-related complications as safety outcomes. We tried to include randomized controlled trials (RCTs) and non-randomized studies (including non-RCTs, interrupted time series, controlled before-and-after studies, cohort studies, and case-control studies), if available. Pooled odds ratios (ORs) were calculated and the certainty of evidence was assessed using Grading of Recommendations Assessment, Development, and Evaluation (GRADE) methodology.

**Results:**

Seven studies involving 4,095 patients were included. In the primary outcome, pre-hospital TQ (PH-TQ) use significantly decrease mortality rate in patients with extremity trauma (odds ratio [OR], 0.48, 95% confidence interval [CI] 0.27–0.86, *I*^2^ = 47%). Moreover, the use of PH-TQ showed the decreasing trend of utilization of blood products, such as packed red blood cells (mean difference [MD]: -2.1 [unit], 95% CI: -5.0 to 0.8, *I*^2^ = 99%) or fresh frozen plasma (MD: -1.0 [unit], 95% CI: -4.0 to 2.0, *I*^2^ = 98%); however, both are not statistically significant. No significant differences were observed in the lengths of hospital and intensive care unit stays. For the safety outcomes, PH-TQ use did not significantly increase risk of amputation (OR: 0.85, 95% CI: 0.43 to 1.68, *I*^2^ = 60%) or compartment syndrome (OR: 0.94, 95% CI: 0.37 to 2.35, *I*^2^ = 0%). The certainty of the evidence was very low across all outcomes.

**Conclusion:**

The current data suggest that, in the pre-hospital settings, PH-TQ use for civilian patients with vascular traumatic injury of the extremities decreased mortality and tended to decrease blood transfusions. This did not increase the risk of amputation or compartment syndrome significantly.

**Supplementary Information:**

The online version contains supplementary material available at 10.1186/s13017-024-00536-9.

## Background

In emergency situations, tourniquets (TQ) are used to control severe bleeding by applying pressure on a limb or body part to restrict blood flow to the area. However, while it is useful intervention for controlling extremity hemorrhage [1–3], prolonged application poses inherent risks of complications [4, 5]. In the past, TQs were primarily utilized in military settings; however, recently, this intervention has been increasingly adopted in civilian settings [6–8].

The latest United States national guidelines for field triage of injured patients incorporate the identification of active bleeding, referring TQ as one of the criteria for recognizing a high risk of serious injury [9]. Surgical intervention is commonly indicated for patients who require TQs, and a higher mortality rate is observed in cases where timely TQ placement is not implemented [10]. However, only a few studies have investigated the efficacy of TQs in civilian settings. In military environments, where immediate life-saving measures are often crucial and medical resources may be limited, TQs are more readily employed as first-line interventions for severe extremity bleeding. In contrast, healthcare providers in civilian settings may have a more nuanced approach, aiming to balance the need for hemorrhage control with the potential risks and associated complications. In addition, the threshold for TQ use in civilian settings may be influenced by local protocols and the expertise of the healthcare providers involved. Several studies have not reported survival benefits associated with the use of pre-hospital TQs [11–13]. Therefore, we aimed to perform a systematic review to investigate the effectiveness of pre-hospital TQ application in patients with traumatic vascular injuries of the extremities.

## Materials and methods

### Eligibility criteria and outcome measures

This systematic review was conducted and reported in accordance with the checklist of PRISMA (Preferred Reporting Items for Systematic Reviews and Meta-Analyses) [14]. We included studies that examined the impact of pre-hospital TQ (PH-TQ) utilization on survival outcomes in patients with vascular extremity injuries caused by trauma in a civilian setting. PH-TQ refer to pneumatic or mechanical TQ applied to patients before hospital arrival, while no PH-TQ use was defined as a TQ applied after hospital arrival (late TQ) or no TQ use at all.

The study types included randomized controlled trials (RCTs) and non-randomized studies, which comprised non-RCTs, interrupted time series, controlled before-and-after studies, cohort studies, and case-control studies. Unpublished studies, including conference abstracts or articles, letters, editorials, comments, and case reports, were excluded. In this review, the primary outcome was overall mortality, and the secondary outcomes were the volume of infused blood components and possible TQ-related complications. The study protocol was registered in the International Prospective Register of Systematic Reviews (PROSPERO) in 2023 (CRD42023448057).

### Information sources and search strategy

We searched studies in PubMed, Ovid EMBASE, and the Cochrane Central Register of Controlled Trials (CENTRAL). The detailed search strategy is shown in the Supplementary Materials (Supplementary 1). Our search strategy included all years since database inception and all languages, provided that an English-language abstract is available. The final date of this study was set as June 30, 2023. The reference lists of identified studies were checked for additional relevant articles.

### Study selection

Following the removal of duplicates, titles were independently screened by the reviewers (YCK and TYT), and irrelevant results were removed. The process was followed by title and abstract screening, and a full-text assessment was conducted if the article was deemed potentially relevant. Additional reviewers (MJH and TPL) were invited and a discussion was initiated to reach a consensus regarding any concerns encountered during the selection process.

### Data collection process, data items, and quality assessment

After the final set of included articles was determined, a spreadsheet specifically adapted for this review was created to record the data extracted from the articles. The spreadsheet consisted of various details, including author(s), publication year, country, study design, participant characteristics, intervention type, and key outcomes. The risk of bias in individual studies was independently reviewed by two authors (YCK and KWL). The RoB 2 tool was planned to be used to assess the quality of randomized controlled studies and the ROBINS-I tool for nonrandomized controlled studies [15, 16]. The certainty of evidence was assessed using the Grading of Recommendations Assessment, Development, and Evaluation (GRADE) methodology [17].

### Data synthesis and analysis

The treatment effects for dichotomized outcomes were evaluated using the odds ratio (OR); continuous outcomes were summarized as the mean difference (MD). Zero-count cells in the dichotomized outcomes were handled using the Yate’s correction approach, which added 0.5 to the cells containing 0 (zero events) [18]. Meta-analyses were conducted to combine the outcome data, and the weighted means of the ORs and their corresponding 95% confidence intervals (CIs) were calculated using random effects models (DerSimonian-Laird method) [19]. Sensitivity analysis for primary outcome was conducted by performing a meta-analysis using data from nonrandomized studies with adjusted ORs. To clarify the impact of using PH-TQ on secondary outcomes and to understand whether reducing heterogeneity among studies would affect the research findings, another sensitivity analysis using data from studies comparing PH-TQ use and no TQ use was also conducted. For the article providing median and interquartile range (IQR), the mean and standard deviation (SD) was converted using the formula: mean ≈ median, and SD ≈ IQR / 1.35. To assess the statistical heterogeneity across studies, we calculated I^2^ statistics, with I^2^ > 50%, assuming the presence of significant heterogeneity [20, 21]. The analyses were performed using R (version 4.3.0; R Foundation for Statistical Computing, Vienna, Austria), and the meta-analysis was performed using the meta package. A *p*-value < 0.05 was considered statistically significant.

## Result

### Search results, study characteristics, and quality assessment

**Fig. 1:**
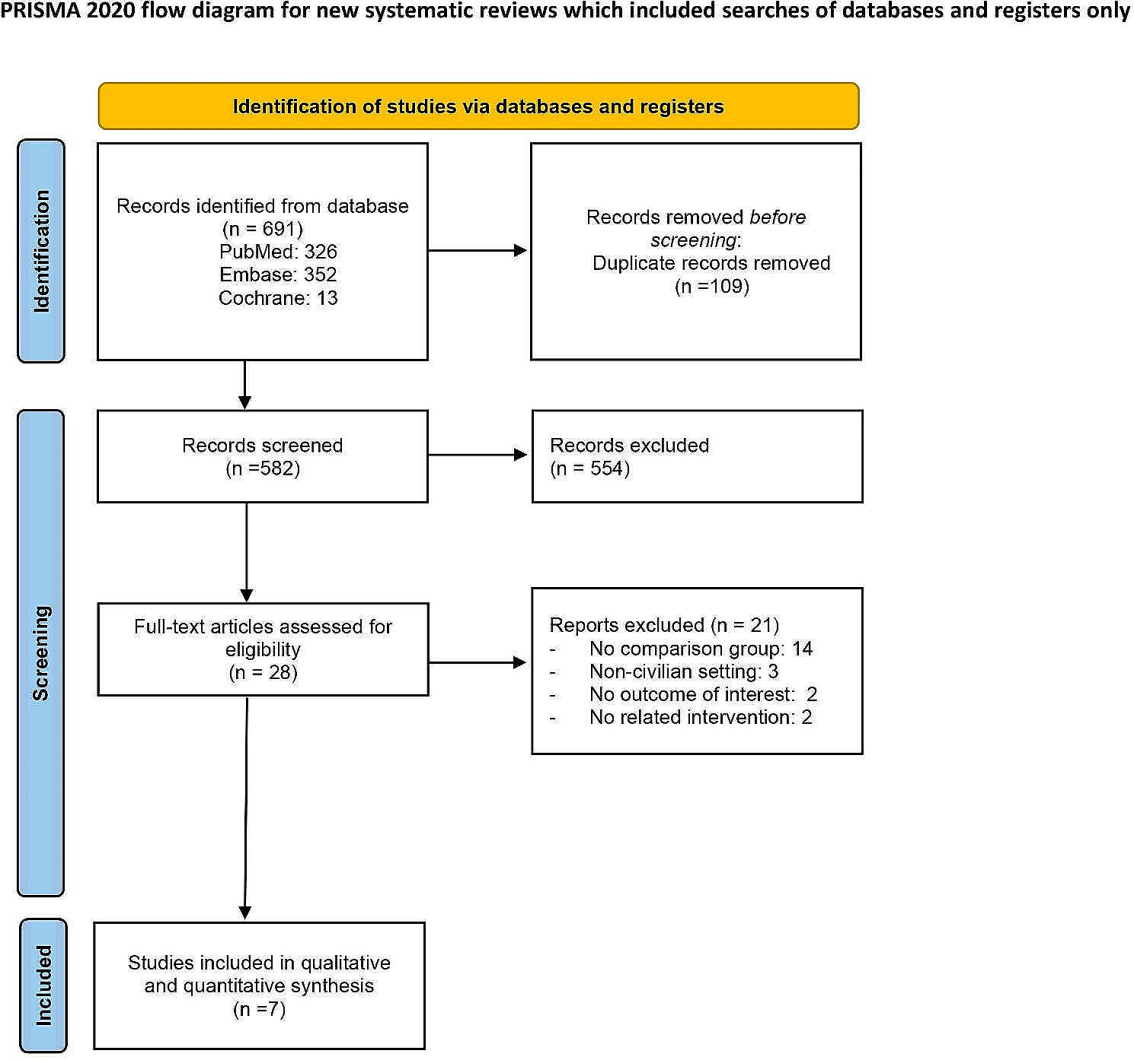
The flow diagram of the systematic review

A flow diagram of the included studies is shown in Fig. 1. After the literature search, 691 records were identified. A total of 109 duplicates and 554 irrelevant articles, were removed after screening the titles and abstracts, resulting to 28 reports for full-text review that were considered potentially relevant. Overall, 7 articles met the inclusion criteria and were included in our study [8, 10–13, 22, 23]. All studies included in our analysis were nonrandomized cohort studies conducted in the North America between 2014 and 2022, comprising six studies from the United States [8, 10–13, 23] and one study from Canada [22]. The reasons for the exclusion of studies were as follows: no comparison group (*n* = 14); for military purpose (*n* = 3); lacks outcome of interest (*n* = 2); and no related intervention (*n* = 2).

**Fig. 2:**
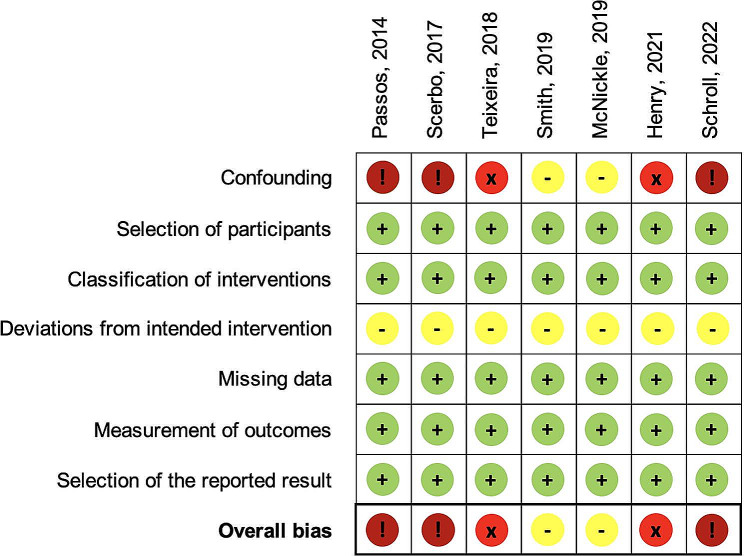
Results of risk of bias assessment for included studies using the ROBINS-I tool

The study characteristics are summarized in Table 1. The analyzed studies involved 4,095 patients, of whom 1,692 and 2,403 received and did not receive PH-TQ, respectively. The studies compared the effect of PH-TQ, with no TQ in five studies [8, 11, 13, 22, 23]; PH-TQ vs. late-TQ in one study [10]; and PH-TQ vs. (late-TQ + no-TQ) in one study [12]. The study participants were mostly males with an average age < 45 years. The participant characteristics in each study are shown in Supplementary 2. All analyzed studies provided crude numbers of mortality, whereas only four [8, 11, 13, 23] controlled for confounders using matching or regression approaches. Three types of blood products were analyzed for infusion: packed red blood cells (pRBCs), platelets, and plasma. Blood transfusion in the first 24 h was reported in five studies [8, 11, 12, 22, 23], blood transfusion in the first hour was reported in one study [10], and the total amount of blood products required in another study [13]. All the analyzed studies documented various side effects following TQ use. These included amputation in all studies [8, 10–13, 22, 23], compartment syndrome in four [10, 11, 13, 22], thromboembolic complication in two [13, 23], nerve palsy in two [12, 13], and infection in two [13, 23]. Individual studies have reported other complications in the pulmonary, cardiac, musculoskeletal, and renal system [11, 13, 23]. The outcomes and potential complications associated with TQ use reported in each study are shown in Supplementary 3. The overall risk of bias varied from moderate to critical, and most of the included studies were subject to a serious to critical risk of bias owing to the risk of confounding factors. The results of the risk of bias assessment are shown in Fig. 2.

**Table 1:** Characteristics of the studies identified in the systematic review

Reference	Year	Country	Study type	Study period	Participants	Intervention	Comparison
Passos et al.	2014	Canada	Retrospective cohort study	01/2001–12/2010	190	PH tourniquet (*n* = 4)	No tourniquet (*n* = 186)
Scerbo et al.	2017	US	Retrospective cohort study	10/2008–01/2016	281	PH tourniquet (*n* = 252)	Late tourniquet (*n* = 29)
Teixeira et al.	2018	US	Retrospective cohort study	01/2011–12/2016	1026	PH tourniquet (*n* = 181)	No tourniquet (*n* = 845)
Smith et al.	2019	US	Retrospective cohort study	2010–2018	204	PH commercial tourniquet application (*n* = 127)	No tourniquet (*n* = 77)
McNickle et al.	2019	US	Retrospective cohort study	01/2013–12/2017	138	PH tourniquet (*n* = 69)	No tourniquet (*n* = 69)
Henry et al.	2021	US	Retrospective cohort study	10/2015–07/2019	944	PH tourniquet (*n* = 97)	No tourniquet (*n* = 847)
Schroll et al.	2022	US	Prospective cohort study	2015–2020	1312	PH improvised or commercial tourniquet application (*n* = 962)	No tourniquet + late tourniquet (*n* = 350)

PH, prehospital; PH tourniquet: tourniquet applied to patients before arriving at a hospital; late tourniquet: tourniquet applied after hospital arrival; no tourniquet: no tourniquet use at all

### Quantitative synthesis

**Fig. 3:**
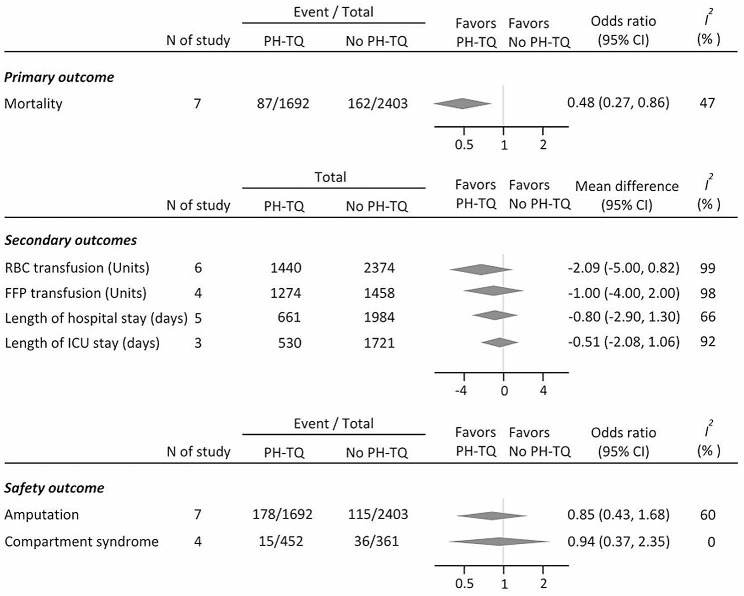
Forest plot of study outcome

The results are summarized in Fig. 3, and detailed information on each outcome is provided in the Supplementary Appendix. For the primary outcome, the pooled results demonstrated applying PH-TQ (compared to those who did not receive PH-TQ) was associated with a significantly lower mortality rate (OR, 0.48; 95% CI 0.27–0.86; I^2^, 47%) (Fig. 3 and Supplementary 4). In the sensitivity analysis of primary outcome, the results remained statistically significant when using only adjusted outcomes in four studies (aOR, 0.34, 95% CI 0.19–0.59, I^2^, 0%) (Supplementary 5). For secondary outcomes, the use of PH-TQ is not associated with reduced use of blood products such as pRBC (MD, -2.09 [unit]; 95% CI -5.00–0.82, I^2^, 99%) or FFP (MD, -1.0 [unit]; 95% CI -4.0–2.0; I^2^, 98%) (Fig. 3, Supplementary 6–7) No significant difference was observed regarding the length of hospital stay (MD, -0.80 [day]; 95% CI -2.90–1.30, I^2^, 66%) or intensive care unit length of stay (MD, -0.51 [day]; 95% CI -2.08–1.06; I^2^, 92%) (Fig. 3, Supplementary 8–9).

For the safety outcomes, the use of PH-TQ is not associated with increased amputation (OR, 0.85; 95% CI 0.43–1.68; I^2^, 60%) and compartment syndrome (OR, 0.94; 95% CI, 0.37–2.35; I^2^, 0%) (Fig. 3, Supplementary 10–11). The sensitivity analysis utilizing data from studies comparing PH-TQ use and no TQ use revealed that, despite a reduction in heterogeneity among studies, the utilization of PH-TQ is not significantly associated with secondary outcomes, including safety outcomes (Supplementary 12–17). The funnel plot, which exhibit asymmetry, is shown in Supplementary 18. Because of the limited number of studies included (*n* < 10), the Egger’s test was not performed. The certainty of evidence (CoE) using the GRADE method for the treatment recommendations of individual endpoints is summarized in Table 2. The CoE was downgraded by domains in the risk of bias and inconsistency owing to the high proportion of studies with a serious risk of bias and high heterogeneity across studies. An asymmetric funnel plot for mortality outcomes was observed. Although only seven studies were included in the review, the publication bias domain was downgraded as concerns arose. In summary, the overall CoE showed very low quality of evidence across all reported endpoints.

**Table 2:** Certainty of evidence (CoE) using the Grading of Recommendations Assessment, Development and Evaluation method to assess the quality of evidence with individual endpoint

No of trials (No of patients)	Risk of bias	Inconsistency	Indirectness	Imprecision	Publication bias	Effect (95% CI)	Overall quality of evidence
**Overall mortality**							
7 (4095)	Downgraded^*^	Not downgraded *I*^2^ = 47%	Not downgraded	Not downgraded	Downgraded^‡^	OR = 0.48 (0.27 to -0.86)	⊕⊖⊖⊖ VERY LOW
**RBC transfusion**							
6 (3787)	Downgraded^*^	Downgraded *I*^2^ = 99%	Not downgraded	Downgraded^†^	NA	MD = -2.09 (-5.00 to 0.82)	⊕⊖⊖⊖ VERY LOW
**FFP transfusion**							
4 (2732)	Downgraded^*^	Downgraded *I*^2^ = 98%	Not downgraded	Downgraded^†^	NA	MD = -1.00 (-4.00 to 2.00)	⊕⊖⊖⊖ VERY LOW
**Length of hospital stays (days)**							
5 (2645)	Downgraded^*^	Downgraded *I*^2^ = 66%	Not downgraded	Downgraded^†^	NA	MD = -0.80 (-2.90 to 1.30)	⊕⊖⊖⊖ VERY LOW
**Length of hospital stays (days)**							
3 (2251)	Downgraded^*^	Downgraded *I*^2^ = 92%	Not downgraded	Downgraded^†^	NA	MD = -0.51 (-2.08 to 1.06)	⊕⊖⊖⊖ VERY LOW
**Amputation**							
7 (4095)	Downgraded^*^	Downgraded *I*^2^ = 60%	Not downgraded	Downgraded^†^	NA	OR = 0.85 (0.43 to 1.68)	⊕⊖⊖⊖ VERY LOW
**Compartment syndrome**							
4 (813)	Downgraded^*^	Not downgraded *I*^2^ = 0%	Not downgraded	Downgraded^†^	NA	OR = 0.94 (0.37 to 2.35)	⊕⊖⊖⊖ VERY LOW

CI: Confidence interval; OR, odds ratio; MD, mean difference; NA: non-available

^*^Concerns arose because more than half of the included studies had a serious to critical overall risk of bias; therefore, the certainty of the evidence was downgraded by one level

^†^Imprecision due to confidence intervals, as 95% CI crosses MD of 0 and OR of 1; therefore, certainty of evidence was downgraded by 1 level

^‡^Concerns arose due to possible presence of publication bias led by asymmetry funnel plot; therefore, certainty of evidence was downgraded by 1 level

**GRADE Working Group grades of evidence: High certainty**: We are very confident that the true effect lies close to that of the estimate of the effect. **Moderate certainty**: We are moderately confident in the effect estimate. The true effect is likely to be close to the estimate of the effect; however, there is a possibility that it is substantially different **Low certainty**: Our confidence in the effect estimate is limited. The true effect may be substantially different from the estimate of the effect. **Very low certainty**: We have very little confidence in the effect estimate. The true effect is likely to be substantially different from the estimate of effect

## Discussion

This systematic review identified seven cohort studies investigating the benefits of PH-TQ use in patients with vascular extremity injuries and demonstrated that early placement of TQ in the pre-hospital setting may confer a survival benefit for patients with vascular injuries in the extremities. The analysis did not reveal any significant differences in the transfusion of blood products, ICU and hospital lengths of stay, or the occurrence of potential complications associated with the intervention. Substantial heterogeneity was noted across the pooled effect estimates.

A previous meta-analysis conducted by Latina et al., which included four articles comparing the clinical effectiveness of pneumatic or mechanical tourniquets, did not find conclusive evidence regarding the impact of emergency PH-TQ placement on overall mortality and utilization of blood products [24]. Moreover, three included studies reported adjusted results on overall mortality [11, 13, 23]. Only two studies employing case-control matching of participants were included in the meta-analysis [11, 13], and one study was excluded because of unclear reporting of the effect estimate [23]. For comparison, the meta-analysis in our study was conducted using ORs as the effect measures. Although not all crude data were provided in a case-control manner, we assumed that the severity of the intervention group would not be less serious than that of the control group. In the sensitivity analysis, four of seven included studies that provided adjusted ORs (aOR) were used for the meta-analysis. The result was away from null, and the heterogeneity statistic I^2^ decreased from 47 to 0%, as we estimated the effect. Furthermore, our study used patients who did not receive PH-TQ, including no TQ application and hospital TQ application, as our control group, which was different from the previous study. We pooled the controls because vascular extremity trauma cases are regarded as time-sensitive [25] and adverse outcomes might occur when the intervention is delayed, possibly even when it is administered upon arrival at hospitals [10]. For component therapy, the heterogeneity across the studies was high, and different variable measurements (e.g., blood products needed in the first hour and first day, or the total amount of blood products needed), were reported. Our study did not identify any supporting evidence indicating that the use of PH-TQs led to a reduction in the need for blood product transfusion. Each study identified in the systematic review reported different TQ-related adverse events. However, the diagnosis of complications has not been clearly reported across studies, making comparisons difficult. Although some of the included studies utilized various statistical methods to address confounding factors, most of them were subject to serious or critical risks, and co-intervention in addition to TQ use may have led to bias. The inherent limitations associated with the retrospective cohort study design may constrain the interpretation of the present meta-analytical findings, highlighting the need for a well-designed clinical trial of better quality.

The utilization of PH-TQ varies within each emergency medical service (EMS) system and its use is influenced by local protocols. A previous study conducted in the United States reported an estimated incidence of 0.2 PH-TQ applications per 1000 EMS activations [7], and this trend was increasing, especially in urban areas [6]. However, although the idea of using TQ is becoming more prevalent in specific communities, we should be aware that the procedure may result in complications and that not every TQ is being used properly [26]. Extremity injuries encompass a wide spectrum ranging from severe deformities to subtle injuries that can be challenging to diagnose. Based on limited experience in using PH-TQ, the importance of adequate training for healthcare providers or first responders and more consistent protocols cannot be overemphasized. Furthermore, in trauma cases, time plays a critical role because the timing of interventions and treatments can substantially influence patient outcomes. It is crucial for practitioners not only to possess the skills and knowledge to correctly apply TQ but also recognize the importance of swiftly using them.

The adverse events reported in the included studies did not show a significant increase in complications related to the use of TQs, and none of the studies reported a direct association with the interventions. Adverse events may have contributed to the injury itself rather than the use of TQs. The severity of injuries tended to be lower in civilian settings than in military settings [27]. It was anticipated that pre-hospital stays for civilians would be shorter and that the likelihood of complications resulting from prolonged TQ application would be reduced. However, both civilian and military settings were composed mostly of young male-injured individuals; the potential complications to the older population or special population needed to be deliberate, considering that these populations are prone to cardiovascular or other adverse events.

Our review has several limitations. First, we included only a limited number of available studies, and no RCTs were identified. This limitation may have affected the overall strength of the evidence and ability to draw solid conclusions. However, it is not possible to perform RCTs in the real-world. To avoid confounding factors, we performed a sensitivity analysis using aORs and led to the same conclusion. Furthermore, cases of TQ application may be deemed more serious, and the efficacy might be underestimated, as all of the reported aORs in the included studies consistently deviated significantly from the null value. Second, we did not compare our method with other hemostasis methods such as manual arterial compression, hemostatic agents, or other hemostatic medical devices. Future studies should incorporate comparative analyses to provide a more comprehensive evaluation of the effectiveness of an intervention. In addition to hemostatic devices, comprehensive approaches for managing traumatic injuries are required. Third, the study did not incorporate time-related factors, such as the time of injury, time to arrival at the hospital, or time to tourniquet application. Time plays a crucial role in trauma cases because the timing of interventions and treatments can significantly affect patient outcomes. Hence, a standardized trauma registry should be established. In addition to data collection and longitudinal follow-up, the registry can serve as a tool for quality improvement initiatives, strengthening each component of acute care for trauma cases.

## Conclusion

The meta-analysis revealed that early placement of a TQ in the pre-hospital setting may provide a survival advantage for patients with vascular injuries in the extremities and decrease the use of blood products. There was no increase in the risk of amputation or compartment syndrome. Further large-scale prospective studies are needed to verify these findings.

### Electronic supplementary material

Below is the link to the electronic supplementary material.

Supplementary Material 1: Supplementary 1. Search strategy. Supplementary 2. Characteristics of participants in included studies. Supplementary 3. Outcomes and potential complications related to tourniquet use in included studies. Supplementary 4. Forest plot of overall mortality of pre-hospital tourniquets vs. no pre-hospital tourniquets. Supplementary 5. Sensitivity analysis for overall mortality of pre-hospital tourniquets versus no pre-hospital tourniquets. Supplementary 6. Forest plot of mean difference in red blood cell transfusion of prehospital tourniquets vs. no pre-hospital tourniquets. Supplementary 7. Forest plot of mean difference in fresh frozen plasma transfusion of pre-hospital tourniquets vs. no pre-hospital tourniquets. Supplementary 8. Forest plot of mean difference in length of hospital stay of prehospital tourniquets vs. no pre-hospital tourniquets. Supplementary 9. Forest plot of mean difference in intensive care unit length of stay with pre-hospital tourniquets vs. no pre-hospital tourniquets. Supplementary 10. Forest plot of amputation with pre-hospital tourniquet vs. no prehospital tourniquet. Supplementary 11. Forest plot of compartment syndrome in the pre-hospital tourniquet vs. no pre-hospital tourniquet group. Supplementary 12. Funnel plot for the included studies

## Data Availability

All data generated or analysed during this study are included in this published article and its supplementary information files.
