# Supplementary material for: Effectiveness and safety of tourniquet utilization for civilian vascular extremity trauma in the pre-hospital settings: a systematic review and meta-analysis
Source: World J Emerg Surg. 2024 Mar 19;19:10. doi: 10.1186/s13017-024-00536-9 (PMC10949629; doi:10.1186/s13017-024-00536-9)
Supplement: Supplementary file 1 — Supplementary Material 1: Supplementary 1. Search strategy. Supplementary 2. Characteristics of participants in included studies. Supplementary 3. Outcomes and potential complications related to tourniquet use in included studies. Supplementary 4. Forest plot of overall mortality of pre-hospital tourniquets vs. no pre-hospital tourniquets. Supplementary 5. Sensitivity analysis for overall mortality of pre-hospital tourniquets versus no pre-hospital tourniquets. Supplementary 6. Forest plot of mean difference in red blood cell transfusion of prehospital tourniquets vs. no pre-hospital tourniquets. Supplementary 7. Forest plot of mean difference in fresh frozen plasma transfusion of pre-hospital tourniquets vs. no pre-hospital tourniquets. Supplementary 8. Forest plot of mean difference in length of hospital stay of prehospital tourniquets vs. no pre-hospital tourniquets. Supplementary 9. Forest plot of mean difference in intensive care unit length of stay with pre-hospital tourniquets vs. no pre-hospital tourniquets. Supplementary 10. Forest plot of amputation with pre-hospital tourniquet vs. no prehospital tourniquet. Supplementary 11. Forest plot of compartment syndrome in the pre-hospital tourniquet vs. no pre-hospital tourniquet group. Supplementary 12. Funnel plot for the included studies [file 13017_2024_536_MOESM1_ESM.pdf]

SUPPLEMENTARY MATERIAL

Effectiveness and Safety of Tourniquet Utilization for Civilian Vascular Extremity Trauma in the Pre-hospital Settings: A Systematic Review and Meta-analysis

Supplementary 1. Search strategy

PubMed

("emergency medical services"[MeSH Terms] OR ("prehospital"[All Fields] OR "prehospitally"[All Fields])) AND ("tourniqueted"[All Fields] OR "tourniquets"[MeSH Terms] OR "tourniquets"[All Fields] OR "tourniquet"[All Fields]) AND ("civilian"[All Fields] OR "civilians"[All Fields] OR ("layperson"[All Fields] OR "laypersons"[All Fields]) OR ("wounds and injuries"[MeSH Terms] OR (("extremities"[MeSH Terms] OR "extremities"[All Fields] OR "limb"[All Fields]) AND ("injuries"[MeSH Subheading] OR "injuries"[All Fields] OR "trauma"[All Fields] OR "wounds and injuries"[MeSH Terms] OR ("wounds"[All Fields] AND "injuries"[All Fields]) OR "wounds and injuries"[All Fields] OR "trauma s"[All Fields] OR "traumas"[All Fields])) OR (("extremities"[MeSH Terms] OR "extremities"[All Fields] OR "extremity"[All Fields] OR "extremity s"[All Fields]) AND ("injuries"[MeSH Subheading] OR "injuries"[All Fields] OR "trauma"[All Fields] OR "wounds and injuries"[MeSH Terms] OR ("wounds"[All Fields] AND "injuries"[All Fields]) OR "wounds and injuries"[All Fields] OR "trauma s"[All Fields] OR "traumas"[All Fields])) OR "vascular system injuries"[MeSH Terms]))

Ovid EMBASE

('emergency care'/exp OR 'acute care' OR 'acute medical care' OR 'emergency care' OR 'emergency health care' OR 'emergency medical care' OR 'patient care, prehospital' OR 'pre-hospital care' OR 'pre-hospital patient care' OR 'prehospital care' OR 'prehospital patient care' OR prehospital) AND ('civilian'/exp OR civilian OR 'layperson'/exp OR 'limb injury'/exp OR 'limb injury' OR 'injury'/exp OR 'blood vessel injury'/exp OR 'blood vessel damage' OR 'blood vessel injury' OR 'blood vessel lesion' OR 'blood vessel trauma' OR 'injury, vascular' OR 'vascular accident' OR 'vascular accident syndrome' OR 'vascular damage' OR 'vascular injuries' OR 'vascular injury' OR 'vascular system injuries' OR 'vascular system injury' OR 'vascular trauma') AND ('tourniquet'/exp OR 'clampease disc' OR 'hysynal' OR 'arm leg tourniquet' OR 'arm/leg tourniquet, reusable' OR 'arm/leg tourniquet, single-use' OR 'external fixator vascular compressor' OR 'external vascular compressor' OR 'finger/toe tourniquet,

sterile' OR 'non-latex tourniquet cuff bandage' OR 'reprocessed tourniquet cuff' OR 'reusable arm/leg tourniquet' OR 'single-use arm/leg tourniquet' OR 'single-use tourniquet cuff' OR 'sterile finger tourniquet' OR 'sterile finger/toe tourniquet' OR 'sterile toe tourniquet' OR 'tourniquet' OR 'tourniquet bandage' OR 'tourniquet cuff' OR 'tourniquet cuff bandage, non-latex' OR 'tourniquet cuff, reprocessed' OR 'tourniquet cuff, reusable' OR 'tourniquet cuff, single-use' OR 'tourniquet strap device' OR 'tourniquet, device (physical object)' OR 'tourniquets' OR 'vascular compressor, external') AND [embase]/lim

Cochrane Central Register of Controlled Trials (CENTRAL)

#1 MeSH descriptor: [Emergency Medical Services] explode all trees
#2 prehospital
#3 civilian
#4 layperson or laypeople
#5 MeSH descriptor: [Wounds and Injuries] explode all trees.
#6 limb injury
#7 extremity injury
#8 MeSH descriptor: [Vascular System Injuries] explode all trees
#9 tourniquet
#10 MeSH descriptor: [Tourniquets] explode all trees.
#11 #1 OR #2
#12 #3 OR #4 OR #5 OR #6 OR #7 OR
#13 #8 OR #9
#14 #10 AND #11 AND #12

Supplementary 2. Characteristics of participants in included studies

Author (Year)	Participants	PH-TQ	No PH-TQ	Age (years) Mean (SD)		Sex (male) n (%)		ISS Mean (SD)		Extremity AIS Mean (SD)		Pre-hospital SBP (mmHg) Mean (SD)		Prehospital HR (bpm) Mean (SD)		Pre-hospital GCS Mean (SD)	
				PH-TQ	Non PH-TQ	PH-TQ	Non PH-TQ	PH-TQ	Non PH-TQ	PH-TQ	Non PH-TQ	PH-TQ	Non PH-TQ	PH-TQ	Non PH-TQ	PH-TQ	Non PH-TQ
Passos (2014)*	190	4	186	41 (12)	36 (16)	4 (100%)	156 (83.9%)	17 (7)	16 (10)	-	-	99 (34)	127 (30)	99 (27)	103 (26)	11 (7)	15 (1)
Scerbo (2017)†	281	252	29	33 [25-46]	34 [24-50]	212 (84.1%)	27 (93.1%)	9 [5-17]	20 [9-27]	3 [2-3]	3 [3-4]	119 [92-139]	100 [83-113]	100 [84-120]	122 [87-135]	15 [14-15]	14 [3-15]
Teixeira (2018)*	1026	181	845	34.4 (14.7)	35.9 (13.8)	157 (87.2%)	708 (83.8%)	13.2 (10.3)	11.3 (8.4)	36 (20%)‡	77 (9.1%)‡	125.3 (94.2)	121.7 (34.3)	105.9 (28.7)	92.6 (27.4)	28 (15.7%)‡	91 (10.9%)‡
Smith (2019)	204	127	77	31.3 (7.89)	31.2 (14.04)	111 (87.4%)	68 (88.3%)	9 (5.63)	10.1 (5.26)	2.8 (2.25)	2.7 (1.75)	114 (22.54)	98 (35.1)	100 (22.54)	104 (43.87)	-	-
McNickle (2019)*	138	69	69	35.0 (12.46)	36.3 (13.29)	56 (81.1%)	53 (76.8%)	13.1 (6.65)	12.3 (7.48)	3.2 (0.83)	3 (0.83)	126 (33.23)	130 (24.92)	110 (33.23)	100 (24.92)	-	-
Henry (2021)	944	97	847	34.8 (13.3)	36.8 (12.4)	83 (85.6%)	712 (84.1%)	13.4 (8.1)	13.7 (7.3)	24 (24.7%)‡	86 (10.2%)‡	113 (45.4)	119 (33.2)	101 (18.3)	96 (15.8)	14 (1.3)	13 (2.5)

Schroll (2022)	1312	962	350	36.6 (15.51)	36.6 (14.97)	819 (85.1%)	297 (84.9%)	10.1 (9.30)	9.3 (9.35)	2.4 (1.24)	2.2 (0.94)	120 (31.02)	121 (37.42)	99 (31.02)	96 (37.42)	-	-

* Physiologic status on arrival in the trauma room.

† presented with median (IQR). IQR, interquartile range

‡ presented with n (%) of AIS>=4 or GCS <=8.

PH, pre-hospital; TQ, tourniquet; SBP, systolic blood pressure; HR, Heart rate; GCS, Glasgow Coma Scale; AIS, Abbreviated Injury Scale; ISS, Injury Severity Score; SD, standard deviation

Supplementary 3. Outcomes and potential complications related to tourniquet use in included studies

Author	Year	Primary outcome	Transfusion-related variables	Adverse effect related variables	Healthcare-related parameters	Adjustment
Passos et al.	2014	Overall mortality	PRBC transfusion in 24 h<br>FFP transfusion in 24 h<br>Platelet transfusion in 24 h	Compartment syndrome<br>Amputation	Hospital length of stay	None
Scerbo et al.	2017	Overall mortality	PRBC transfusion in 1 h<br>Plasma transfusion in 1 h<br>Platelet transfusion in 1 h	Amputation<br>Compartment syndrome<br>Vascular injury	Hospital length of stay<br>ICU length of stay	None
Teixeira et al.	2018	Overall mortality	Massive transfusion<br>PRBC transfusion in 24 h<br>FFP transfusion in 24 h<br>Platelet transfusion in 24 h	Delayed amputation<br>Thromboembolic complication<br>Pulmonary complication<br>Cardiac complication<br>Infectious complication	Hospital length of stay<br>ICU length of stay<br>Ventilator use	Patient demographics, physiologic and injury-related parameters.
Smith et al.	2019	Overall mortality	Total RBCs transfusion<br>Total FFPs transfusion	Secondary amputation<br>Nerve palsy<br>Local infection<br>Compartment syndrome<br>Fasciotomy<br>Deep vein thrombosis	Hospital length of stay	Patient demographics and injury severity.

McNickle et al.	2019	Overall mortality	Blood transfusion in 24 h<br>PRBC transfusion in 24 h<br>Intravenous fluid volume in the ED	Initial amputation<br>Delayed amputation<br>Rhabdomyolysis<br>Compartment syndrome<br>Acute kidney injury	Hospital-free days<br>ICU-free days<br>Ventilator-free days	Patient demographics, injured artery, ISS, and mechanism.
Henry et al.	2021	Overall mortality	PRBC transfusion in 4 and 24 h	Delayed amputation	Hospital length of stay<br>ICU length of stay	Physiologic parameters, mechanism, traumatic amputations, ISS, prehospital transport time.
Schroll et al.	2022	Overall mortality	PRBC transfusion in 24 h<br>FFP transfusion in 24 h	Amputation<br>Nerve palsy	None	None

PRBC, pack red blood cell; FFP, fresh frozen plasma; ISS, injury severity score; ED, emergency department; ICU, intensive care unit

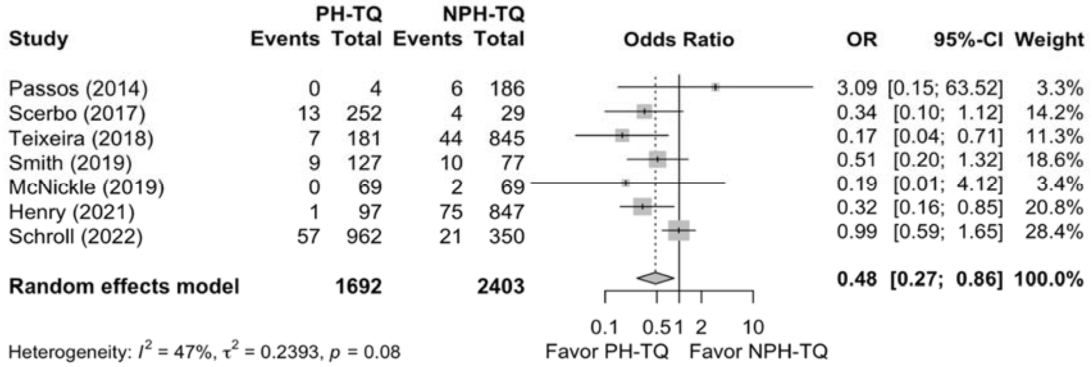

Supplementary 4. Forest plot of overall mortality of pre-hospital tourniquets vs. no pre-hospital tourniquets. PH-TQ, pre-hospital tourniquet; NPH-TQ, no pre-hospital tourniquet.

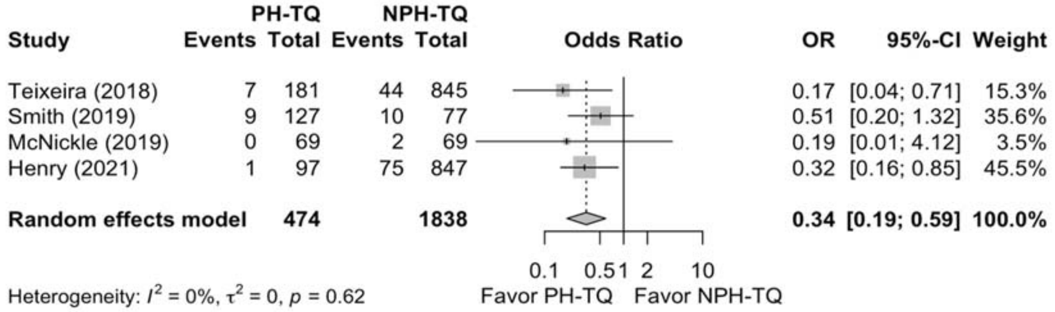

Supplementary 5. Sensitivity analysis for overall mortality of pre-hospital tourniquets versus no pre-hospital tourniquets. PH-TQ, pre-hospital tourniquet; NPH-TQ, no pre-hospital tourniquet.

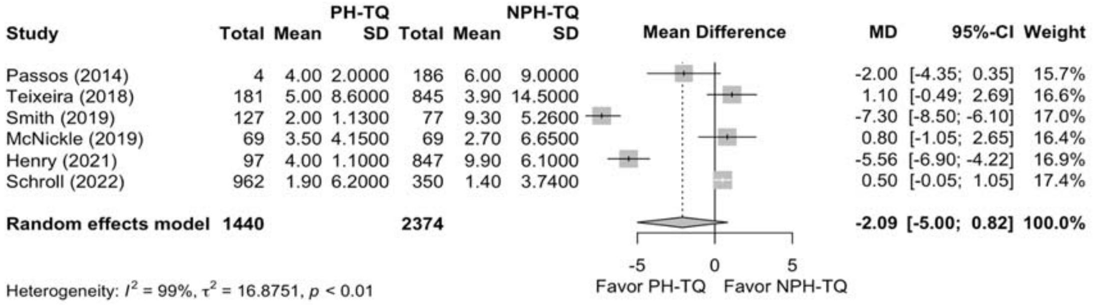

Supplementary 6. Forest plot of mean difference in red blood cell transfusion of pre-hospital tourniquets vs. no pre-hospital tourniquets. PH-TQ, pre-hospital tourniquet; NPH-TQ, no pre-hospital tourniquet.

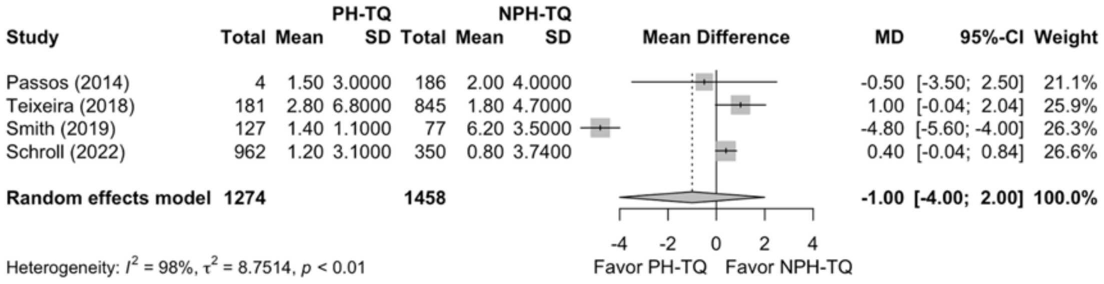

Supplementary 7. Forest plot of mean difference in fresh frozen plasma transfusion of pre-hospital tourniquets vs. no pre-hospital tourniquets. PH-TQ, pre-hospital tourniquet; NPH-TQ, no pre-hospital tourniquet.

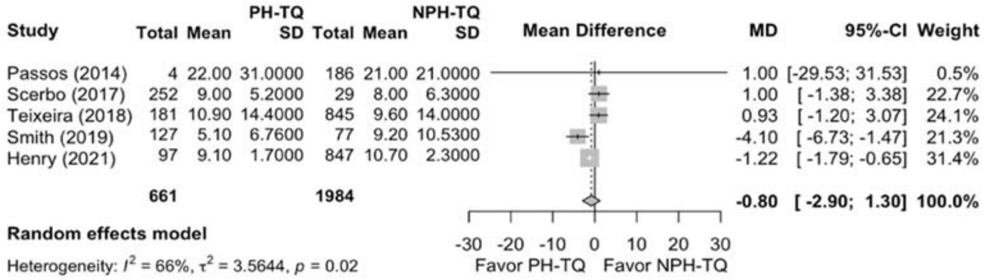

Supplementary 8. Forest plot of mean difference in length of hospital stay of pre-hospital tourniquets vs. no pre-hospital tourniquets. PH-TQ, pre-hospital tourniquet; NPH-TQ, no pre-hospital tourniquet.

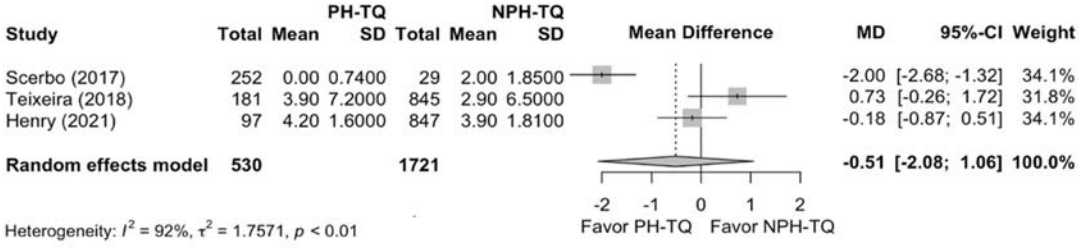

Supplementary 9. Forest plot of mean difference in intensive care unit length of stay with pre-hospital tourniquets vs. no pre-hospital tourniquets. PH-TQ, pre-hospital tourniquet; NPH-TQ, no pre-hospital tourniquet.

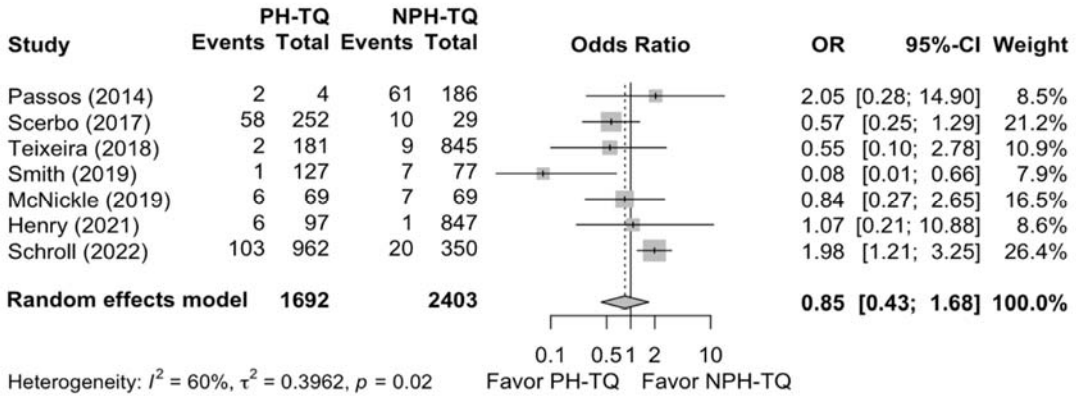

Supplementary 10. Forest plot of amputation with pre-hospital tourniquet vs. no pre-hospital tourniquet. PH-TQ, pre-hospital tourniquet; NPH-TQ, no pre-hospital tourniquet.

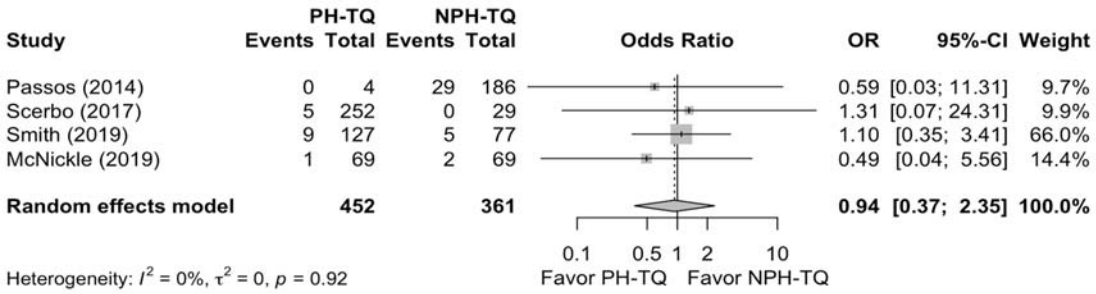

Supplementary 11. Forest plot of compartment syndrome in the pre-hospital tourniquet vs. no pre-hospital tourniquet group. PH-TQ, pre-hospital tourniquet; NPH-TQ, no pre-hospital tourniquet.

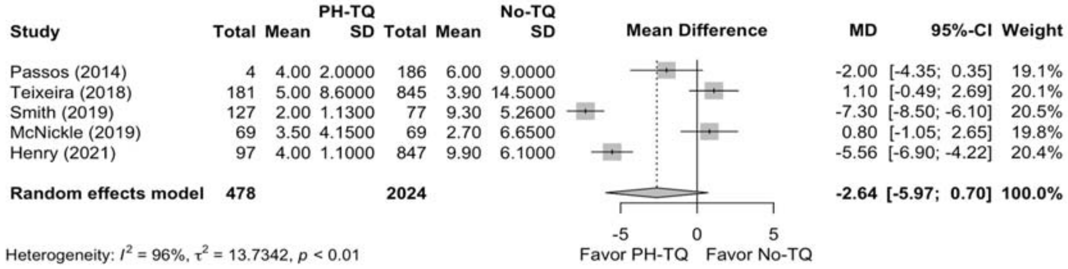

Supplementary 12. Forest plot of mean difference in red blood cell transfusion of pre-hospital tourniquets vs. no tourniquets. PH-TQ, pre-hospital tourniquet; No-TQ, no tourniquet.

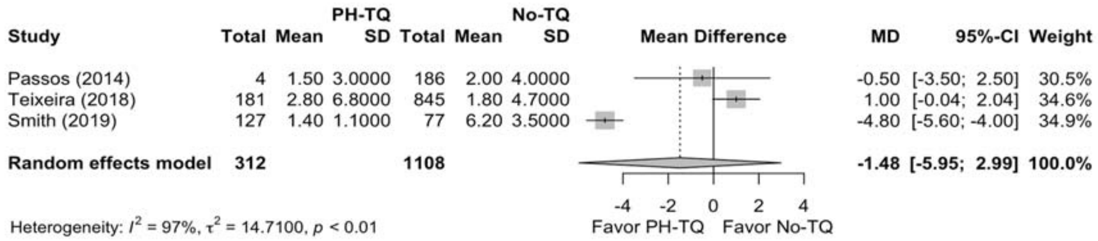

Supplementary 13. Forest plot of mean difference in fresh frozen plasma transfusion of pre-hospital tourniquets vs. no tourniquets. PH-TQ, pre-hospital tourniquet; No-TQ, no tourniquet.

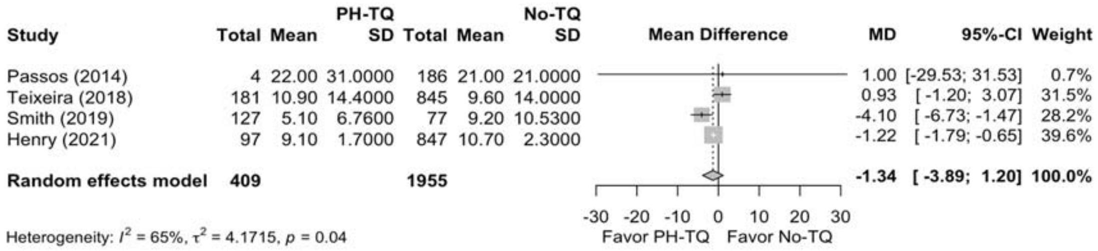

Supplementary 14. Forest plot of mean difference in length of hospital stay of pre-hospital tourniquets vs. no tourniquets. PH-TQ, pre-hospital tourniquet; No-TQ, no tourniquet.

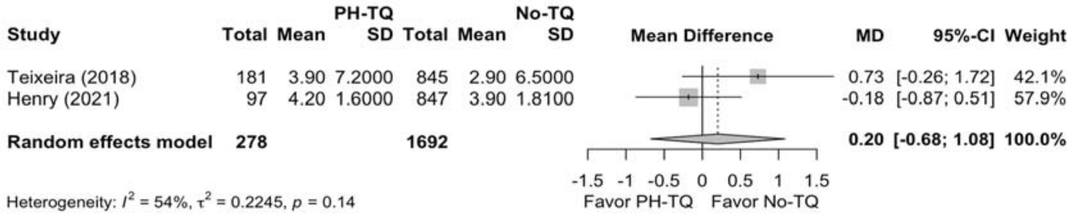

Supplementary 15. Forest plot of mean difference in intensive care unit length of stay with pre-hospital tourniquets vs. no tourniquets. PH-TQ, pre-hospital tourniquet; No-TQ, no tourniquet.

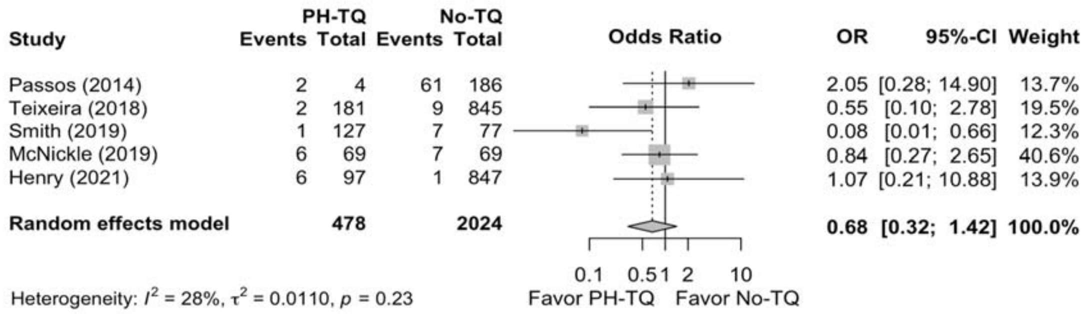

Supplementary 16. Forest plot of amputation with pre-hospital tourniquets vs. no tourniquets. PH-TQ, pre-hospital tourniquet; No-TQ, no tourniquet.

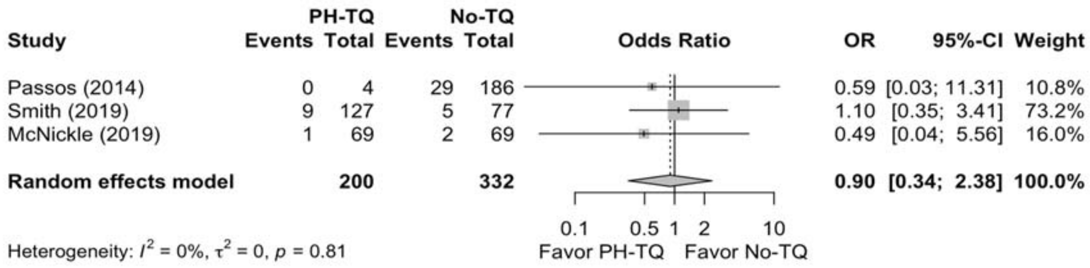

Supplementary 17. Forest plot of compartment syndrome in the pre-hospital tourniquets vs. no tourniquets. PH-TQ, pre-hospital tourniquet; No-TQ, no tourniquet.

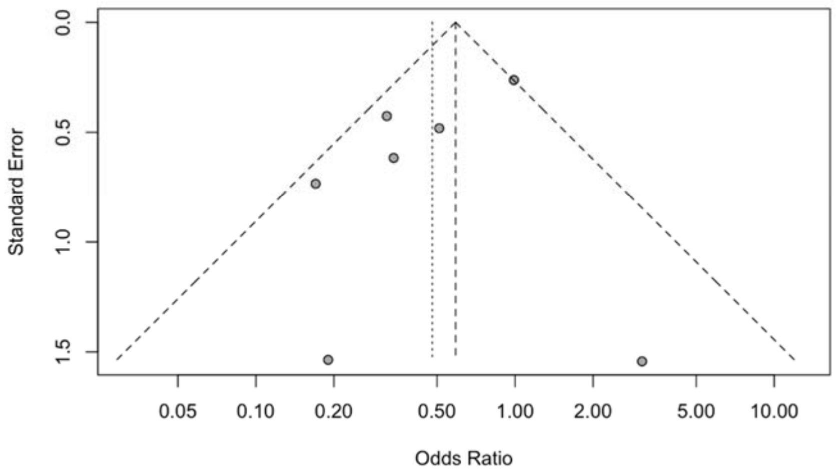

Supplementary 18. Funnel plot for the included studies.
